# First *in situ* evidence of wakes in the far field behind offshore wind farms

**DOI:** 10.1038/s41598-018-20389-y

**Published:** 2018-02-01

**Authors:** Andreas Platis, Simon K. Siedersleben, Jens Bange, Astrid Lampert, Konrad Bärfuss, Rudolf Hankers, Beatriz Cañadillas, Richard Foreman, Johannes Schulz-Stellenfleth, Bughsin Djath, Thomas Neumann, Stefan Emeis

**Affiliations:** 1 University of Tuebingen, ZAG, Environmental Physics, 72074 Tuebingen, Germany; 2 Karlsruhe Institute of Technology (KIT), Institute of Meteorology and Climate Research (IMK-IFU), 82467 Garmisch-Partenkirchen, Germany; 3 Technische Universität Braunschweig, Institute of Flight Guidance, 38108 Braunschweig, Germany; 4 UL DEWI - UL International GmbH, 26382 Wilhelmshaven, Germany; 5 Helmholtz-Zentrum Geesthacht (HZG), Institute of Coastal Research, 21502 Geesthacht, Germany

## Abstract

More than 12 GW of offshore wind turbines are currently in operation in European waters. To optimise the use of the marine areas, wind farms are typically clustered in units of several hundred turbines. Understanding wakes of wind farms, which is the region of momentum and energy deficit downwind, is important for optimising the wind farm layouts and operation to minimize costs. While in most weather situations (unstable atmospheric stratification), the wakes of wind turbines are only a local effect within the wind farm, satellite imagery reveals wind-farm wakes to be several tens of kilometres in length under certain conditions (stable atmospheric stratification), which is also predicted by numerical models. The first direct *in situ* measurements of the existence and shape of large wind farm wakes by a specially equipped research aircraft in 2016 and 2017 confirm wake lengths of more than tens of kilometres under stable atmospheric conditions, with maximum wind speed deficits of 40%, and enhanced turbulence. These measurements were the first step in a large research project to describe and understand the physics of large offshore wakes using direct measurements, together with the assessment of satellite imagery and models.

## Introduction

Offshore wind farms contribute a considerable fraction to the production of renewable electrical energy. In 2015, 12 GW of offshore wind-energy capacity was successfully installed in Europe^1^. In Germany offshore capacity is expected to reach 7.8 GW by 2020^2^. In Europe, it is expected to reach 73 GW by 2030^3^. A significant number of these new installations will be in the North and Baltic Seas^4,5^.

**Figure 1:**
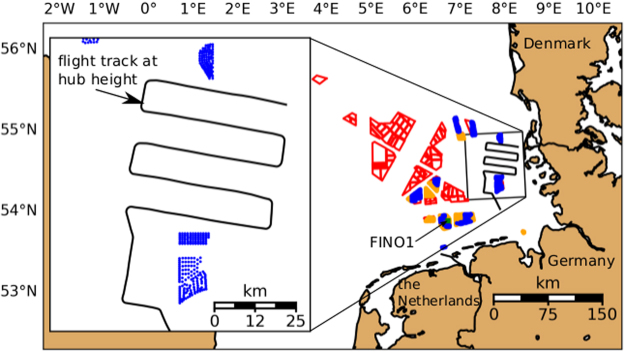
Distribution of offshore wind farms in the German Bight. Blue regions are farms currently in operation and orange regions are those wind farms that are under construction or have been approved (as of 2017). Red polygons indicate farms with a submitted application (as of 2016). The plot on the left side indicates the flight track of Flight 7 on September 10, 2016. The blue dots represent the location of the individual wind turbines.

**Figure 2:**
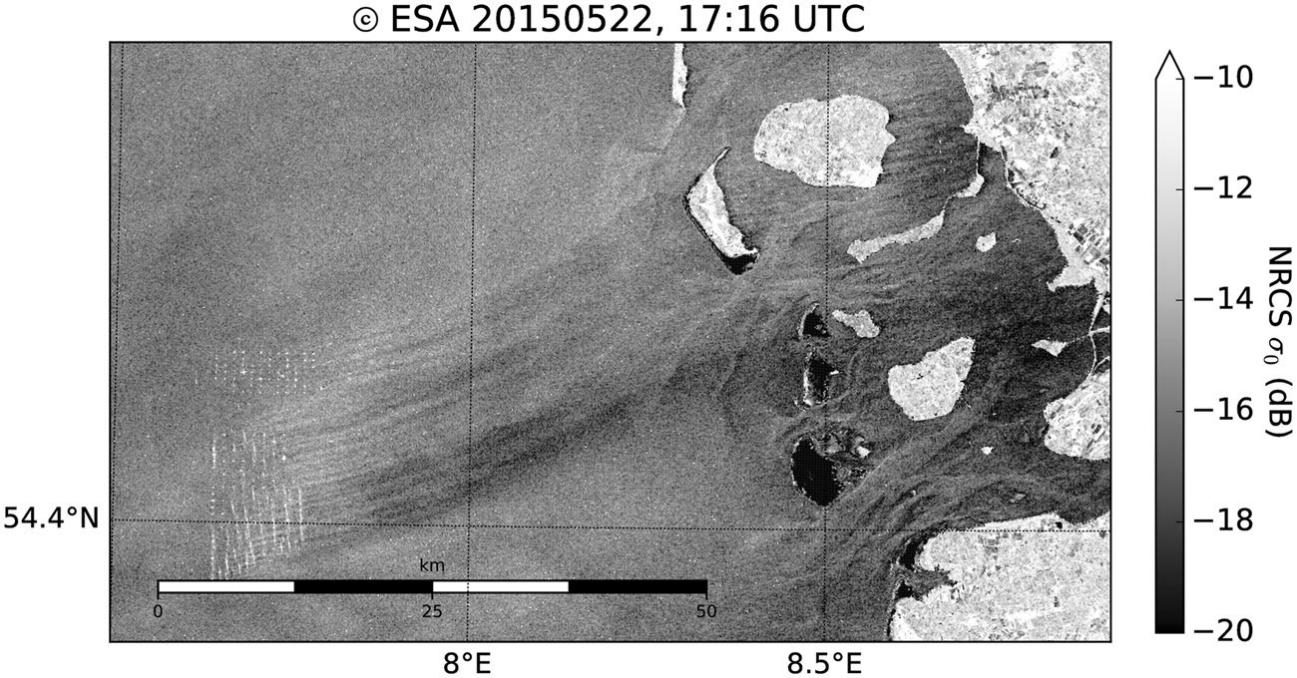
Example of a SENTINEL-1A satellite SAR image (Copernicus Sentinel data [2015]) acquired over the North Frisian Coast in the German Bight on May 22, 2015 at 17:16 UTC with westerly winds created by Matplotlib^37^. The white dots on the lower left are radar signatures from windfarm turbines of the three wind parks Amrumbank West, Nordsee Ost and Meerwind Süd/Ost. A wake of reduced wind speed generated by the wind turbines is indicated by darker streaks downwind of the wind farms.

For an optimal use of the marine areas^6^, wind farms are constructed at favourable locations and in clusters (see Fig. 1). As wind farms are built to extract considerable kinetic energy from the atmosphere, a downwind wake region is formed, characterised by a reduced mean wind speed and, additionally, an enhanced level of turbulence. Most research in this area focuses on wakes behind single turbines, and on the wake interaction from a larger number of turbines within one and the same wind farm^7^. Only some experimental and recent numerical studies consider the wakes of entire wind farms and the impact of wakes on neighbouring downwind wind farms on a larger spatial scale^6,8–22^. The spatial extension of wakes from offshore wind farms is not understood to the extent that the length of a wake may be predicted based on all influencing parameters, such as wind-farm characteristics, atmospheric conditions, and sea state^23^. The most efficient mechanism for wake recovery is the vertical transfer of momentum from higher atmospheric layers downwards^24^, implying atmospheric turbulence to be the decisive parameter governing wake recovery^16,25,26^. Atmospheric turbulence is primarily produced from vertical wind speed gradients (mechanical turbulence) and thermal convection (thermal turbulence). Over rough land surfaces, both mechanical and thermal turbulence are abundant and wakes are usually short (at maximum a few kilometres in length). Much less turbulence is produced at sea, because of the small surface friction and weak temperature gradients, since the response of the ocean to solar radiation is slow. The wakes from wind farms over the sea are, therefore, expected to extend further downwind than over land, especially under a stably stratified flow, which inhibits thermally produced turbulence^5,27^. Since offshore wind farms are located close to the coastline (i.e. a distances less than 100 km to the coast), warm air from land may flow over the colder sea to generate stable stratification, especially during spring and summer. While not yet verified by direct *in situ* measurements, analytical^20,24,28^ and numerical flow models^13,22,29,30^ predict the length of far wakes up to 100 km in stable stratification. Further, satellite images from synthetic aperture radar (SAR) suggest the existence of wake lengths of several tens of kilometres (Fig. 2) under stable atmospheric conditions, i.e., in the absence of thermally produced turbulence^31,32^. However, such images are rare as the repeat cycle of the satellite is about 11–12 days and lack some observational verification in addition.

Verification of numerical and analytical models and SAR is difficult because *in situ* measurements of offshore wind-farm wakes only exist in the near field, directly behind single turbines and wind farms^22,33,34^. In fact, *in situ* measurements of far-field wakes at hub height on a larger scale behind whole offshore wind farms are not currently available. The German research project WIPAFF (WInd PArk Far Field)^35^ has performed the first aircraft measurements of the far wakes of wind farm clusters in the North Sea. We summarise the first measurements here and compare them with numerical simulations of the Weather Research and Forecasting model (WRF)^36^.

## Methods

**Figure 3:**
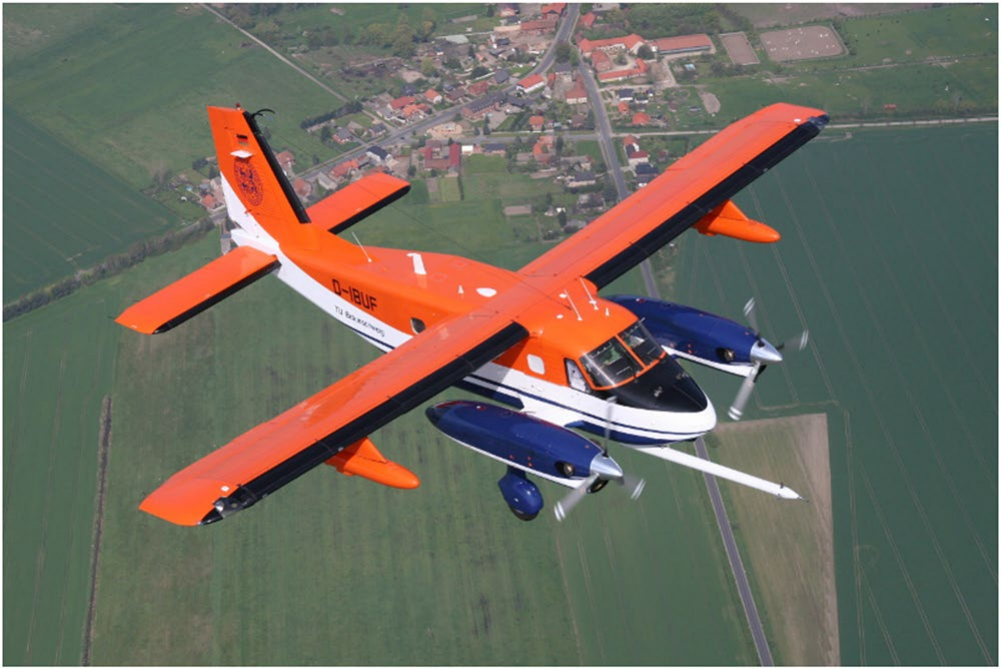
The research aircraft Dornier DO-128 of the Technische Universität Braunschweig.

Table 1 gives an overview of all 41 measurement flights performed during the WIPAFF project with the Dornier DO 128 aircraft (Fig. 3) in 2016 and 2017 over the German Bight. The starting points of all flights were Wilhelmshaven, Borkum or Husum airport. The aircraft airspeed during the measurements was 66 m s^−1^.

**Table 1:** Full list of all measurement flights conducted within the WIPAFF project.

Flight code	Date (dd.mm.yyyy)	Start Time (UTC)	End Time (UTC)	WS (m s^−1^)	Wind dir (*°*)	Wake length (km)	Atmospheric stratification
September							
Flight 1	06.09.2016	14:13	17:20	7	190	25	stable
Flight 2	07.09.2016	09:25	13:00	4	210	20	stable
Flight 3	07.09.2016	10:00	14:00	4	190	at least 10	stable
Flight 4	08.09.2016	10:38	14:25	8	120	at least 40	stable
Flight 5	09.09.2016	10:54	14:50	6	240	at least 45	stable
Flight 6	09.09.2016	15:43	19:17	6	250	at least 5	unstable
**Flight 7**	**10.09.2016**	**07:30**	**11:30**	**7**	**190**	**45**	**stable**
Flight 8	10.09.2016	12:05	16:00	4	190	at least 20	stable
March–April							
Flight 1	30.03.2017	15:57	19:02	15	240	70	stable
Flight 2	31.03.2017	15:36	19:00	13	180	50	stable
Flight 3	05.04.2017	15:42	16:34	14	310	10	neutral
Flight 4	06.04.2017	15:29	18:22	8	310	at least 10	unstable
Flight 5	09.04.2017	12:36	16:07	7	220	at least 50	stable
Flight 6	09.04.2017	16:32	20:12	4	200	n.a.	stable
Flight 7	11.04.2017	11:25	15:10	8	300	5	unstable
Flight 8	11.04.2017	16:12	20:04	8	240–280	25	neutral
Flight 9	13.04.2017	13:35	17:39	16	290	10	neutral
May–June							
Flight 1	17.05.2017	12:35	16:28	8	110	n.a.	stable
Flight 2	17.05.2017	17:16	21:22	12	120	55	stable
Flight 3	23.05.2017	15:42	16:34	5	250	at least 25	stable
Flight 4	23.05.2017	13:18	17:15	11	310	at least 35	neutral
Flight 5	24.05.2017	07:40	11:34	8	300	n.a.	unstable
Flight 6	24.05.2017	12:13	16:11	9	270	5	unstable
Flight 7	27.05.2017	09:57	13:58	10	150	at least 50	stable
Flight 8	27.05.2017	14:39	18:36	12	140	55	stable
Flight 9	31.05.2017	09:58	13:46	8	290	2	unstable
Flight 10	31.05.2017	15:00	18:50	9	290	0	unstable
Flight 11	01.06.2017	08:55	12:54	6	300	0	unstable
Flight 12	02.06.2017	08:55	12:40	4	170	at least 15	stable
August							
Flight 1	08.08.2017	10:35	14:35	9	80	at least 35	stable
Flight 2	08.08.2017	15:06	19:07	14	80	at least 55	stable
Flight 3	09.08.2017	10:34	14:37	15	210	n.a.	unstable
Flight 4	09.08.2017	15:09	19:05	13	240	n.a.	unstable
Flight 5	10.08.2017	12:49	16:54	5	330	n.a.	unstable
Flight 6	14.08.2017	12:08	16:07	8	150	at least 35	neutral
Flight 7	14.08.2017	16:40	20:31	7	120	50	stable
Flight 8	15.08.2017	09:22	13:15	8	180	30	stable
Flight 9	17.08.2017	08:06	12:10	12	160	40	stable
October							
Flight 1	14.10.2017	14:59	18:40	15	260	n.a.	stable
Flight 2	15.10.2017	09:05	13:09	14	200	n.a.	unstable
Flight 3	15.10.2017	13:52	17:50	13	190	at least 25	stable

Wake length: Assessed wake distance with a wind speed deficit with more than 0.1 m s^−1^ compared to the undisturbed flow. Wake lengths measured during a flight pattern that did not cover the full extent of the wake are indicated with “at least”. Some flights focused on the processes above wind farms, hence, no data is available describing the length of the wakes, for such flights the wake length is not available (n. a.). Atmospheric stratification: Estimation of the atmospheric stability by analysing the airborne measured potential temperature vertical profiles between near surface (30 m) and hub height (100 m), which were flown close to the wind farm. WS means wind speed. Bold text marks the investigated flight in this study.

### Meteorological data

**Figure 4:**
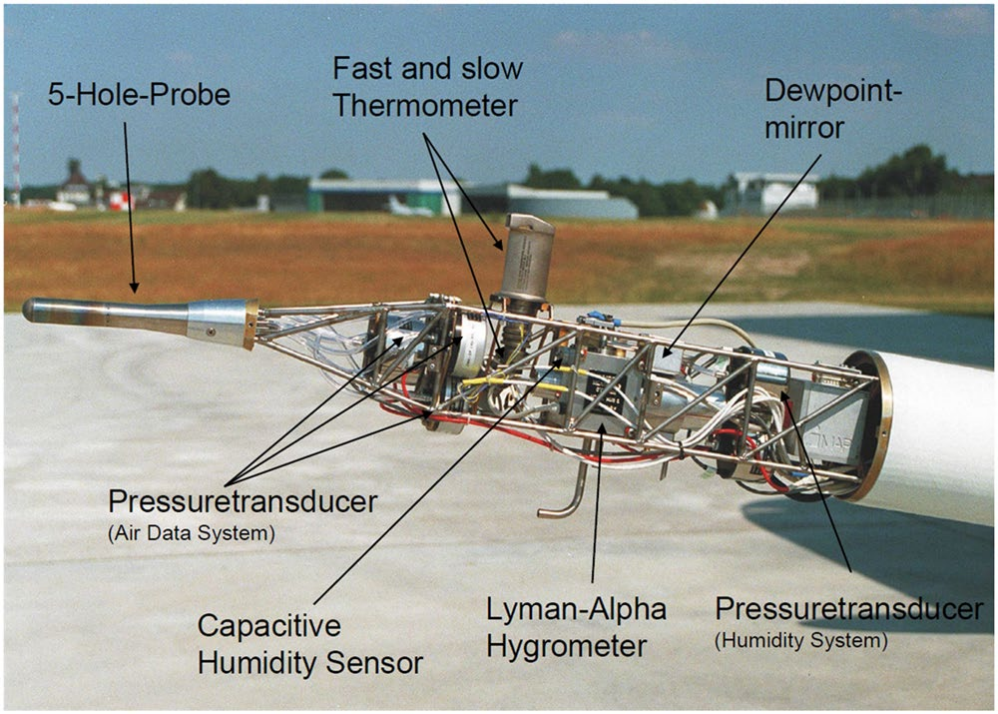
Instrumentation of the nose boom of the DO-128.

The wind vector measurement is performed by measuring the flow speed and flow angles at the aircraft nose with a multi-hole flow probe (Figs 3 and 4), as well as the aircraft’s motion and orientation in the geodetic coordinate system with an inertial measurement unit (IMU) and the ground speed vector with a combination of IMU and GPS. More details on the aircraft’s sensor system can be found in^38–40^. The total duration of a measurement flight lasted 2 to 4 h, and the main downwind flight pattern lasted about 1 h as shown in Fig. 1. The data acquisition rate is 100 Hz. Given the information of these sensors, the wind speed can be calculated as

1 $${\bf{u}}={{\bf{v}}}_{{\bf{gs}}}+{\bf{M}}({{\bf{v}}}_{{\bf{tas}}}+{\rm{\Omega }}\times {\bf{s}}),$$

where **u** is the wind speed vector, **v**_**gs**_ is the ground speed vector, **v**_**tag**_ is the airspeed vector, **M** is the rotation matrix from the aircraft’s fixed coordinate system with respect to the geodetic coordinate system, and **s** is the lever arm between the IMU and the flow probe. The rate of angular rotation vector Ω contains the angular velocities of the aircraft fixed coordinate system relative to the geodetic coordinate system, and is among the primary output data of the IMU. A detailed description of the airborne wind speed measurement, including an error estimation, can be found in^33^ and^41^.

The turbulent kinetic energy, TKE is calculated by

2 $${\rm{T}}{\rm{K}}{\rm{E}}=\frac{1}{2}({\sigma }_{u}^{2}+{\sigma }_{v}^{2}+{\sigma }_{w}^{2})$$

with *σ*_*u*_ representing the fluctuations of the wind vector component *u*, *σ*_*v*_ of the component *v* and *σ*_*w*_ of *w*.

For example, *σ*_*u*_ is computed as

3 $${\sigma }_{u}^{2}=\frac{1}{N-1}\sum _{n=1}^{N}{(u(n)-\bar{u})}^{2},$$

where *N* is the number of data points within the moving data window and $\overline{u}$ denotes the average of *u* within the window. To study the variability of the wind speed field and TKE, it is necessary to determine a suitable horizontal length scale over which to compute the mean wind speed and the fluctuation *σ* of the wind components within sub-legs (data windows) along a flight leg. The method is the so-called moving-average method. Given a series of values (the total data point along one flight leg) and a fixed subset size (sub-legs), the first element of the moving average is obtained by taking the average of the initial fixed subset of the time series. The subset is then modified by a forward shift, so that the first value of the series is excluded, while including the next value following the original subset in the series to create a new subset of numbers for averaging. The process is repeated over the entire data series.

However, sub-legs not exceeding the largest eddies in size insufficiently sample the dynamic wind field, causing a systematic error by systematically under- or overestimating the turbulent wind and its standard deviation^42^. This sampling error can be estimated by the expression stated in^43^ and^44^ representing the absolute systematic statistical uncertainty of the standard deviation *σ*_*u*_ related to a single flight leg on which *σ*_*u*_ was calculated,

4 $${\rm{\Delta }}{\sigma }_{u}=2\frac{{L}_{u}}{{P}_{l}}\cdot {\sigma }_{u},$$

where *L*_*u*_ is the integral length scale^45^ of *u* and *P*_*l*_ the averaging length. The *L*_*u*_ can be explained as the correlation time, i.e. the persistence or memory of the turbulent flow^46^. The integral time scale *I*_*u*_ for the wind speed *u* is

5 $${I}_{u}={\int }_{0}^{{\tau }_{1}}d\tau \,\frac{\overline{u^{\prime} (t+\tau )\cdot u^{\prime} (t)}}{\overline{{u^{\prime} }^{2}}}={\int }_{0}^{{\tau }_{1}}d\tau \,\frac{\overline{{{\rm{Cov}}}_{u}(\tau )}}{{\sigma }_{u}^{2}},$$

where Cov_*u*_ represents the covariance of *u*, and is calculated by integration from zero lag to the first zero crossing at *τ*_1_^47^. The transformation into the *L*_*u*_ is carried out by multiplication of the *I*_*u*_ by the aircraft’s ground speed, assuming that Taylor’s hypothesis of frozen turbulence is valid^45^. For example, the integral length scale for the wind speed *u* for Flight 7 is about 90 m. To obtain an error of less than 10% of *σ*_*u*_, the window length should be at least 1800 m according to Eq. 4. We have defined windows of 2-km width using unweighted means, sequentially shifted through the leg by increments of 0.66 m for a sampling rate of 100 Hz and an aircraft ground speed of 66 m s^−1^. As *σ*_*u*_ is about 0.1 m s^−1^ for Flight 7, the error for the measured wind speed *u* is 1%.

### Scanning lidar

We recorded sea surface measurements using a scanning LiDAR-system supported by a navigation grade IMU for registering the measurement points. The effective pulse rate of 22 kHz theoretically provides spatial-point densities of one per metre along, and five per metre perpendicular to, the flight direction for an effective overall measurement rate of about 4.5 kHz. In addition to spatial information, the calibrated echo amplitude is used to compute the reflectance relative to a perpendicular white target at the same distance.

Data have been calculated as the average relative reflectance over 2 s. Fewer measurement points were received within the wake because of the smoother sea surface. In the averaged data set, this resulted in a generally higher reflectance inside the wake caused by more specular reflections.

### Numerical model WRF

We conducted numerical simulations with the Weather Research and Forecasting Model WRF (Version 3.7.1)^36^ using three nested domains with grid size of 15 km, 5 km and 1.7 km. The nesting allows feedback between the nested domains with an update frequency of 20 s for the second domain and 60 s for the first domain. All model domains have 50 vertical levels with a spacing of approximately 40 m at the rotor area. The model top is at 100 hPa (=16 km). The initial and lateral boundary conditions are defined by the European Centre for Medium-Range Weather Forecasts (ECMWF) model operational analysis data at 6-h intervals. The ECMWF data has a grid size of 0.1 degrees (i.e. similar to the grid size of the first domain). The model is initialised at 12 UTC, 9 September 2016 (i.e. 19 h before the first measurements) and integrated for 36 h.

The following parametrizations are used for all domains: The NOAH land surface model^48^, the WRF double-moment 6-class cloud microphysics scheme (WDM6^49^), the Rapid Radiative Transfer Model for the GCM scheme for short- and longwave radiation^50^ and the Mellor-Yamada-Nakanishi-Niino boundary-layer parametrization^51^. The ocean surface roughness is determined by a modified Charnock relation^52^. In contrast to the two innermost domains, the outermost domain uses the Kain-Fritsch cumulus parametrization scheme^53^.

### Wind farm parameterization

The grid size of the numerical model WRF is too large to capture the effect of a single wind farm explicitly. Therefore, we use the wind farm parametrization of Fitch *et al*.^13^, which acts as a momentum sink for the mean flow and as a source of turbulence at the height of the rotor. The wind turbines at the wind farms Amrumbank West (AW), Windpark Meerwind Süd/Ost (WM) and Nordsee Ost (OWPN) have a hub height ranging from 90 m to 95 m and a diameter of 120 m up to 126 m. Therefore, the rotor area of the wind turbines intersects with three model levels. The effects of the wind turbine towers on the atmosphere are neglected.

A wind turbine extracts kinetic energy from the atmosphere, with the total extracted fraction from the atmosphere described by the thrust coefficient *C*_*T*_. Only a fraction of the extracted kinetic energy is converted into electrical energy as quantified by the power coefficient *C*_*P*_. The difference between *C*_*T*_ and *C*_*P*_ stems from electrical and mechanical losses, and the production of non-productive drag. By neglecting the electrically and mechanically induced losses and assuming that all non-productive drag is converted into electrical energy, the difference *C*_*T*_ − *C*_*p*_ describes the amount of kinetic energy that is extracted from the mean flow and then converted into turbulent kinetic energy^13^.

The coefficients *C*_*T*_ and *C*_*p*_ are a function of wind speed and depend on the type of turbine^13^. The three wind farms of interest (AW, WM, OWPN) have two different wind turbine types: At AW and WM, Siemens SWT 3.6–120 offshore turbines are installed whereas at OWPN, Senvion 6.2 wind turbines are used, with nominal powers of 3.6 MW and 6.2 MW, respectively. Since *C*_*T*_ and *C*_*p*_ for these turbines are unavailable to the public, we adapt coefficients from the wind turbine Siemens SWT 3.6–120 onshore, as these are available online (see http://www.wind-turbine-models.com/turbines/646-siemens-swt-3.6–120-onshore). The model underestimates the wind at hub height by up to 1 m s^−1^. Furthermore, the parametrisation of Fitch *et al*.^13^ neglects the dependence of the power and thrust coefficients on the stability of the atmosphere. Therefore, the power and thrust coefficients chosen in the present study are only a suitable first approximation.

### Measurements of wind-farm wakes in the far field

*In situ* observations from fixed platforms like FINO 1 are available, but do not provide the spatial sampling required to study the three-dimensional structure of wakes. The institutes involved in the WIPAFF project were aware of these shortcomings in currently available data sources. Therefore, we collected *in situ* data with the research aircraft Dornier DO-128 belonging to the Technische Universität Braunschweig, Germany. Measurement flights delivered wind speed and direction, turbulence, temperature, humidity, surface-temperature and sea-state data at high resolution (sampling frequency 100 Hz), similarly to campaigns documented in^38,39^. A laser scanner was also integrated into the research aircraft to determine sea-surface properties.

**Figure 5:**
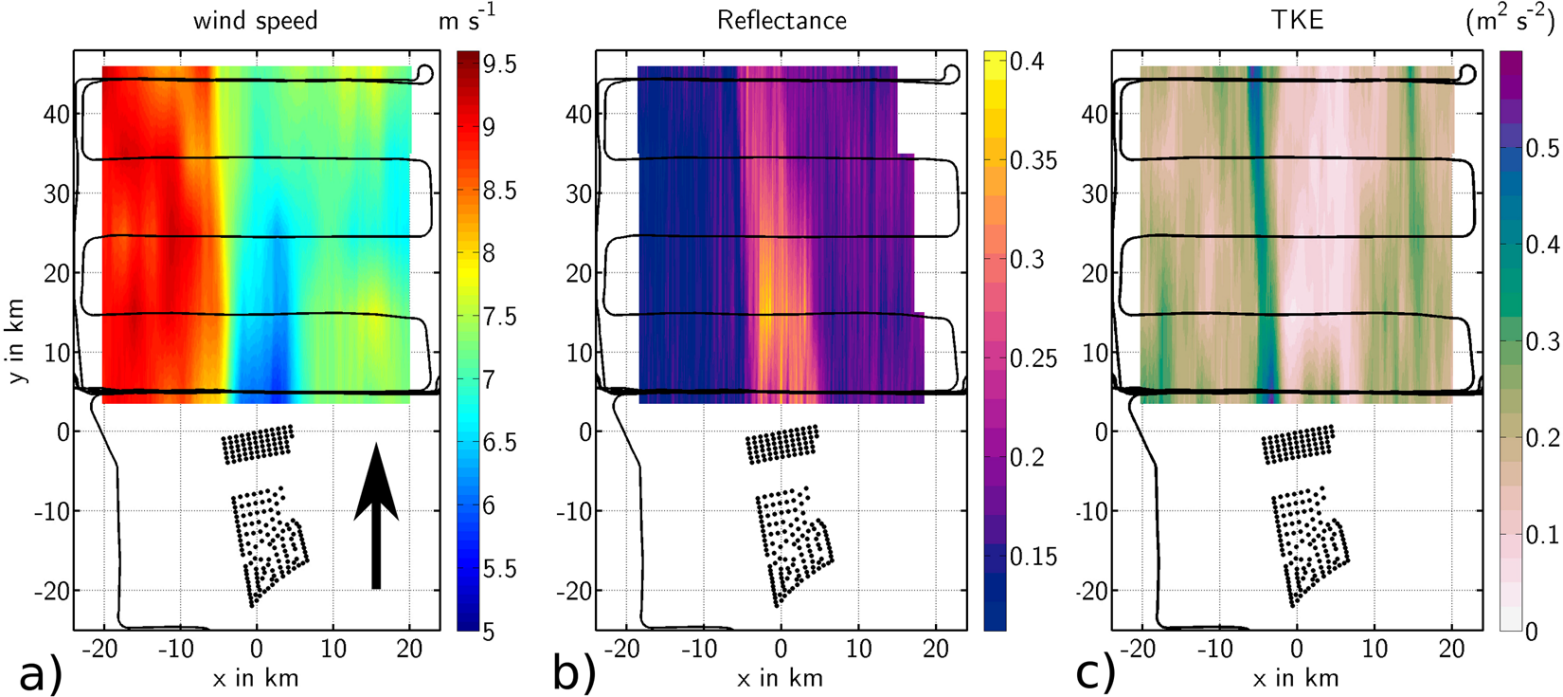
(**a**) Wind speed measurements at hub height (90 m) from the DO 128 flight on September 10, 2016 08:30–09:30 UTC (Flight 7). The wind speed measured along the flight track (black lines) is linearly interpolated perpendicular to the mean wind direction (south 190, indicated by the black arrow). Black dots mark the position of the wind turbines of the wind farms Amrumbank West, Nordsee Ost and Meerwind Süd/Ost. The geographical GPS-coordinates are converted into a Cartesian coordinate system aligned with the mean wind direction (190) for a better comprehension of the orientation and length of the wake. (**b**) As in Fig. 5a), but for the dimensionless reflectance of the sea surface. A higher reflectance may be interpreted as a lower wind speed near the ocean surface. (**c**) As in Fig. 5a), but for the TKE.

We performed 41 measurement flights between September 2016 and October 2017 downwind of wind farm clusters, such as Amrumbank West and Godewind located in the German Bight (Table 1). We discuss the results of Flight 7 on September 10, 2016 here as a typical example for the wake extent during moderate wind speeds of 7–10 m s^−1^ and under stable conditions. Throughout the September 2106 campaign, a dominant high-pressure system was located over Central and Eastern Europe, resulting in the advection of warm sub-tropical air over the German Bight from the south. The warm air over the colder water during the campaign resulted in stable atmospheric stratification (i.e. no thermal turbulence and, therefore, the prevention of convective motion), which is favourable for the generation of long wakes. By vertical profiling of the lower atmosphere with the aircraft, we observed stable conditions over the sea during the September 2016 campaign during 7 flights, where wakes over the whole flight range up to 45 km were detected. In total we detected wakes with a length of at least 10 km during 27 cases, the longest wake length was 70 km (see Table 1). The flight pattern of Flight 7 on September 10 shown in Fig. 5a)–c) measured both the undisturbed air flow and the wake dispersion downwind from the wind farm cluster Amrumbank West, Nordsee Ost and Meerwind Süd/Ost with 90% of the wind turbines running. Several flight legs of 40 km length positioned perpendicular to the mean wind direction and staggered (5, 15, 25, 35, 45 km) behind the wind farm captured both the wake and the adjacent undisturbed air flow at hub height (90 m) of the wind turbines.

Wind speed measurements from Flight 7 are shown in Fig. 5a), where data recorded from individual legs are linearly interpolated, and displayed as coloured contours. Behind the wind farm, a zone of reduced wind speed extended to at least 45 km, with a wind speed deficit up to 3 m s^−1^ at 5 km downwind and about 1 m s^−1^ at 45 km behind the wind farm resulting in a maximum wind speed deficit of 40%. In this manuscript we refer to wind speed deficit as the difference between the flow within the wake and the undisturbed flow outside of the wake on the western side along each flight leg (where the maximum wind speed was measured) instead of using the wind speed measured upstream of the wind farms as a reference. This definition is necessary because of two reasons. First, the wind speed has a gradient from East to West. Therefore, it would be difficult to define an upstream wind speed. Secondly, the upstream wind speed decreased during field experiment. Hence, using the upstream measured wind speed as reference would lead to an underestimation of the wind speed reduction. The wind speed deficit in the wake is aligned along the mean wind direction. The wake sector has the width of the wind farm (10 km) for the closest flight legs (at 5 km and 10 km downwind) and no pronounced spreading out can be detected with increasing distance from the wind farm.

A lower wind speed results in a smoother water surface. The smoothness of the water surface was measured by laser reflectance aboard the aircraft using the downwards-looking laser scanner (Sect. Methods). The scattering of the signal transmitted by the laser is less diffuse for smoother water, hence, the probability of a specular reflection in the direction of the sensor is higher. This effect of increased reflectance at low wind speeds is well known from microwave radar altimeter studies^54^, which we use to help visualise the far wake and relate to SAR images. As shown in Fig. 5b), we measured a higher reflectance by a factor of four inside the wake than in the neighbouring region, indicating lower wind speeds in the wake during Flight 7. *In situ* wind speed measurements (Fig. 5a) and laser reflectance (Fig. 5b) both show a wake throughout the whole scanning area of 45 km downwind of the wind farm. Furthermore, Fig. 5a) and b) display a horizontal wind speed reduction from west to east (i.e. perpendicular to the mean wind direction) caused by the higher surface friction along the coast, east of the flight path.

### Turbulence in the far wake

The degree of atmospheric turbulence impacts the efficiency and fatigue loading of a wind turbine^55^. A typical parameter to describe turbulence is the turbulent kinetic energy (TKE) described in Sect. Methods. Measurements of TKE reveal a far downwind dispersion of the turbulence produced by the wind farm and as a result of the mixing of the wake with the undisturbed flow (Fig. 5c). A slender wake of TKE with a width less than 5 km is aligned with the western edge of the wind farm. A stronger horizontal wind speed gradient exists between the decelerated wind field in the wake and undisturbed wind field to the west. The eastern edge of the wake is much less pronounced as a result of the lower wind speeds along the coast. Inside the wake less turbulence is produced due to a lower wind speed than in the undisturbed flow outside the wake, thus TKE is smaller. Moreover, the eastern boundary of the cluster of wind farms is more irregular compared with the western edge (see Fig. 5c). The TKE of 0.5 m^2^ s^−2^ in the wake sector is about five times that in the undisturbed air flow and decays slowly after 10 km to about 0.3 m^2^ s^−2^. An elevated level of TKE remains at even 45 km downwind of the wind farm. Within the eastern part of the wake, the TKE remains lower (below 0.1 m^2^ s^−2^) than in the undisturbed flow (0.1–0.25 m^2^ s^−2^) at least 45 km downwind on account of the lower wind speeds and reduced horizontal wind shear.

### Comparison with model simulations

**Figure 6:**
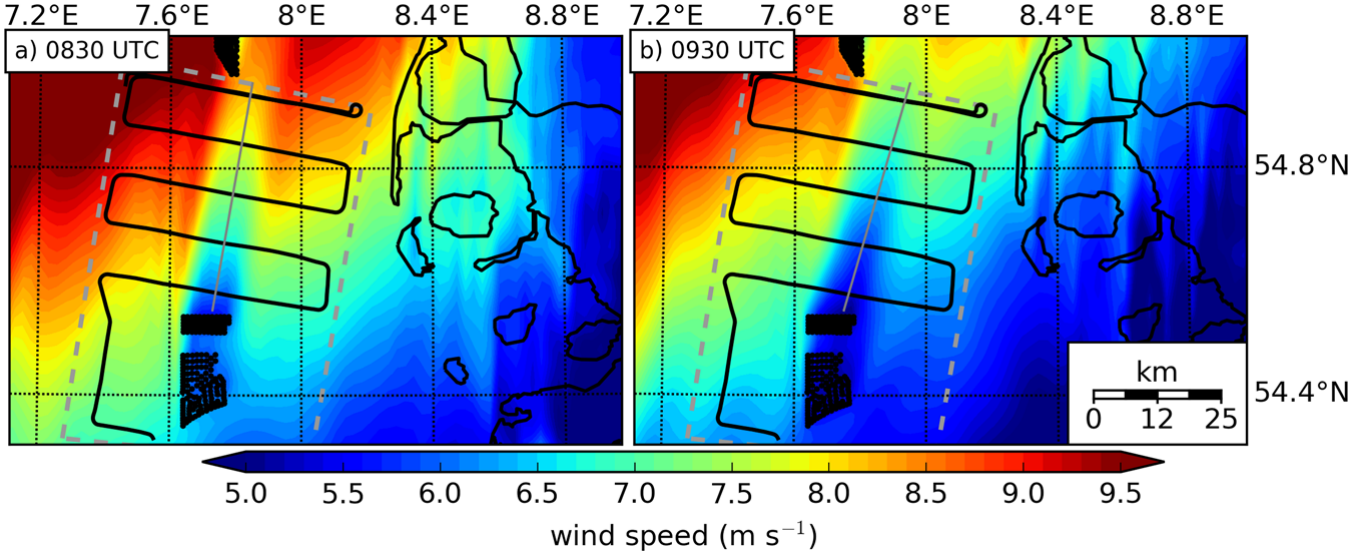
(**a**) WRF model simulation of the wind field at hub height (90 m) for 10 September 2016 08:30 UTC. (**b**) WRF model simulation for 09:30 UTC on the same day. The flight pattern over the German Bight is marked by the black line, the measurement flight domain according to Fig. 5a)–c) by grey dashed line, German and Danish coast by black lines and wind turbines by black dots. Grey line indicates a cross-section of the wind speed, which is displayed in Fig. 7. The figures were generated with Matplotlib^37^.

We performed numerical simulations of the wake using the wind farm parametrisation of Fitch *et al*.^13^ within the Weather Research and Forecasting Model for a grid size of 1.6 km. Operational analysis data from the European Centre for Medium-Range Weather Forecasts (ECMWF) provided the initial and lateral boundary conditions. The model results (Fig. 6) have been obtained for two times at the beginning of the measurement pattern and at the end.

**Figure 7:**
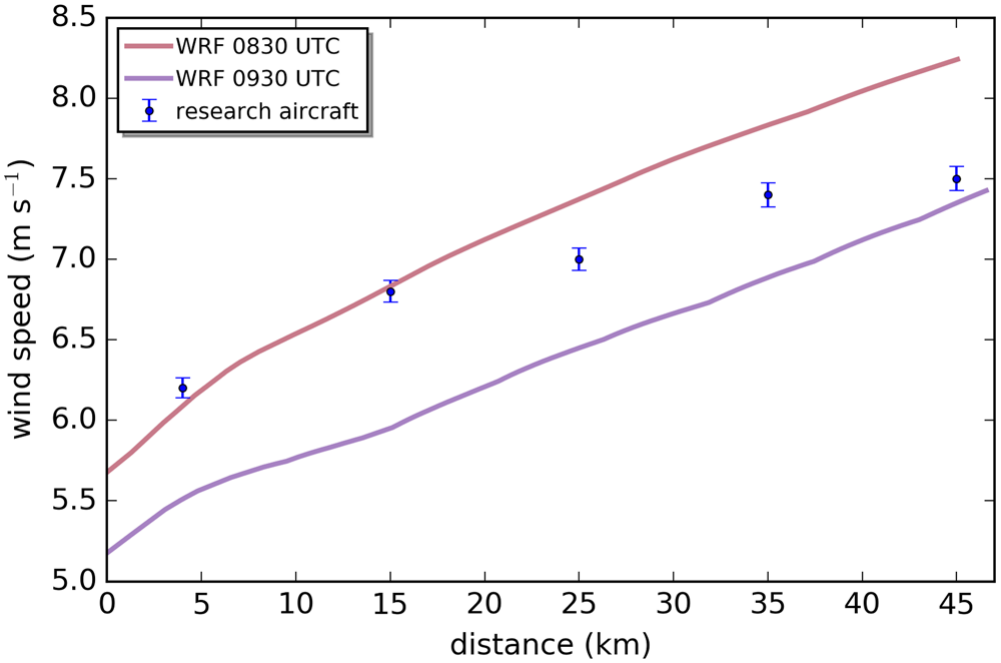
Cross-section along the wake as marked in Fig. 6 of the WRF simulations at 08:30 UTC (red) and 09:30 UTC (purple) and the *in situ* data (blue). Error bars indicate the estimated wind measurement error as explained in Section Meteorological data.

The model simulations reveal a similar structure and orientation of the far wake for the 10 September 2016 as observed by the airborne data (Fig. 6), with a wind speed of about 6 m s^−1^ at the first flight leg 5 km downwind (08:30 UTC) and 7.3 m s^−1^ at the last flight leg (09:30 UTC) 45 km downwind (Fig. 7). However, the observations indicate higher wind speeds within the wake than the simulations (Fig. 7). This finding is consistent with the wind speed observations taken upwind of the wind farm where the model is underestimating the wind speed. Consequently, the wind speed within the wake has to be lower than the *in situ* data, otherwise the wind farm parameterization would underestimate the wind speed deficit induced by the wind farm.

The wind speed averaged over the measurement domain during the flight decreases from approximately 7 m s^−1^ (08:30 UTC) to 5 m s^−1^ (09:30 UTC), which is consistent with flight measurements. The attenuated wind field along the coast observable in the simulations matches well with *in situ* observations (Fig. 5a).

## Discussion

As expected from the results of remote sensing observations, numerical and analytical studies^13,20,22,24,28,30–32,56,57^, the wind speed deficits downwind of offshore wind farms tend to be larger in stable than in unstable conditions, and the lengths of wakes are longer. Likewise, our aircraft measurements show strong indications for longer wakes for all flights under stable situations, whereas wakes were not observed far away from the farms during unstable conditions (see Table 1). These first airborne *in situ* results fortify assumptions from the previous studies. A further detailed analysis of the stratification and wake length will be presented in a future work, as an exact stability analysis is very complex and must be done for each single flight, which is beyond the scope of this paper.

**Figure 8:**
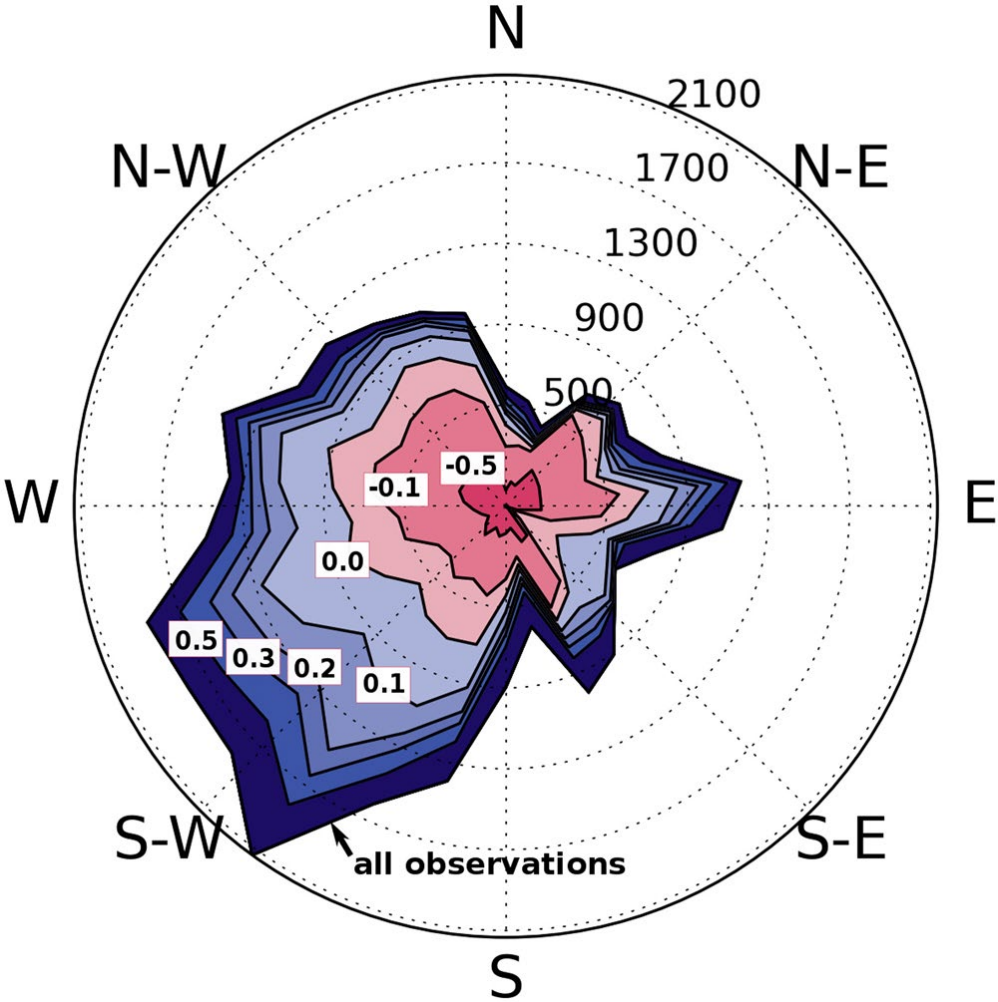
Stability wind rose indicating the frequency (number of 10-min intervals per 12° wind direction sector) of atmospheric stability. Lines are labelled in terms of the stability measure *z*/*L*, where *z* is the height above ground, and *L* is the Monin-Obukhov length. Blue and red shading indicates stable and unstable stratification, respectively. The higher the value the stronger the stability. Data are from the FINO 1 offshore platform in the North Sea for the whole year of 2005 at a height of 60 m above the sea surface. Data is available from http://fino.bsh.de/. Only data with wind speeds between the cut-in (5 m s^−1^) and cut-off (25 m s^−1^) wind speed have been considered.

The question now is how often do stable conditions occur, and are stable conditions coupled to certain wind directions? Fig. 8 displays a stability wind rose (32,736 10-min mean values for the relevant wind speed range of 5 m s^−1^ to 25 m s^−1^) from the offshore research platform FINO 1^58^ located in the German Bight to the north of the island of Borkum (see Fig. 1) for the whole of the year 2005. While 20% of all values exceed a moderate stability of *z*/*L* = 0.2, 10% of all values still exceed a stability of *z*/*L* = 0.5.

Figure 8 also demonstrates a correlation between the wind direction and atmospheric stratification, which is typical for mid-latitudes on the northern hemisphere^59^, resulting from the alternating warm and cold sectors of the eastward moving cyclones at this latitude. Stable situations are most likely found for south-west wind directions, from which we can infer that this is the most likely direction producing long wakes in the North Sea. Further, the predominant wind directions in the North Sea are west and south-west wind directions as 42% of all values in Fig. 8 come from the 90 sector from south to west, meaning we expect stable situations from this predominant sector about 5% of the time. For wind farms located several tens of kilometres downwind of neighbouring wind farms along the main wind direction, the productivity of the downwind farms may be reduced during periods with stable stratification.

Our airborne observations provide the first *in situ* confirmation of the existence of far wakes extending at least 45 km downwind from wind farms, confirming the ability of numerical simulations and SAR satellite images in capturing the spatial structure of wind-farm wakes. Further analysis for different atmospheric conditions are foreseen to provide a clearer quantitative relationship between wind speed, turbulence intensity, atmospheric stability and wake length.
